# *Bacillus cereus* cell response upon exposure to acid environment: toward the identification of potential biomarkers

**DOI:** 10.3389/fmicb.2013.00284

**Published:** 2013-10-02

**Authors:** Noémie Desriac, Véronique Broussolle, Florence Postollec, Anne-Gabrielle Mathot, Danièle Sohier, Louis Coroller, Ivan Leguerinel

**Affiliations:** ^1^ADRIA Développement, UMT 08.3 PHYSI’Opt, QuimperFrance; ^2^EA3882, Laboratoire Universitaire de Biodiversité et Ecologie Microbienne, UMT 08.3 PHYSI’Opt, IFR148 ScInBioS, Université de BrestQuimper, France; ^3^UMR408, Sécurité et Qualité des Produits d’Origine Végétale, Institut National de la Recherche AgronomiqueAvignon, France; ^4^UMR408, Sécurité et Qualité des Produits d’Origine Végétale, Université d’Avignon et des Pays de VaucluseAvignon, France

**Keywords:** *Bacillus cereus*, acid stress response, general stress response, pH homeostasis, metabolic rearrangement, oxidative stress response

## Abstract

Microorganisms are able to adapt to different environments and evolve rapidly, allowing them to cope with their new environments. Such adaptive response and associated protections toward other lethal stresses, is a crucial survival strategy for a wide spectrum of microorganisms, including food spoilage bacteria, pathogens, and organisms used in functional food applications. The growing demand for minimal processed food yields to an increasing use of combination of hurdles or mild preservation factors in the food industry. A commonly used hurdle is low pH which allows the decrease in bacterial growth rate but also the inactivation of pathogens or spoilage microorganisms. *Bacillus cereus* is a well-known food-borne pathogen leading to economical and safety issues in food industry. Because survival mechanisms implemented will allow bacteria to cope with environmental changes, it is important to provide understanding of *B. cereus* stress response. Thus this review deals with the adaptive traits of *B. cereus* cells facing to acid stress conditions. The acid stress response of *B. cereus* could be divided into four groups (i) general stress response (ii) pH homeostasis, (iii) metabolic modifications and alkali production and (iv) secondary oxidative stress response. This current knowledge may be useful to understand how *B. cereus* cells may cope to acid environment such as encountered in food products and thus to find some molecular biomarkers of the bacterial behavior. These biomarkers could be furthermore used to develop new microbial behavior prediction tools which can provide insights into underlying molecular physiological states which govern the behavior of microorganisms and thus opening the avenue toward the detection of stress adaptive behavior at an early stage and the control of stress-induced resistance throughout the food chain.

## INTRODUCTION

*Bacillus cereus* is a Gram positive, facultative anaerobic bacterium belonging to the genus *Bacillus* which may produce endospores. The *B. cereus* group comprises seven recognized species: *B. cereus* and *B. anthracis*, known as human pathogens, *B. thuringiensis* used as biopesticide, *B. mycoides*, *B. pseudomycoides* characterized by rhizoidal formations, *B. weihenstephanensis* including psychrotolerant strains and *Bacillus cytotoxicus* which is the last identified species (Guinebretière et al., 2013). Furthermore, Guinebretière et al. (2008) proposed a division of the *Bacillus cereus *sensu lato into seven major groups (I–VII) using both genetic and phenotypic criteria. Each group corresponds to different virulence potential and to specific thermotypes, showing clear differences in their ability to grow at low or high temperatures (Auger et al., 2008; Guinebretière et al., 2008; Lapidus et al., 2008). Spores and vegetative cells of *B. cereus* are widely encountered in environment such as in soil, considered as its natural habitat, in rhizosphere (Berg et al., 2005), in insects (Luxananil et al., 2001) or mammals (Stenfors Arnesen et al., 2008).

*B. cereus* is a known food-borne human pathogen which frequently causes illnesses. It can cause two types of food poisoning (i) the diarrheic syndrome due to the production in the intestine of enterotoxins such as hemolysin BL (HBL), non-hemolytic enterotoxin (NHE) and CytK (cytotoxin K), and (ii) emetic syndrome due to the production of the cereulide, an emetic toxin produced in food (Lund and Granum, 1997; Lund et al., 2000; Kotiranta et al., 2000). Within the seven groups of *Bacillus sensu lato* the involvement of groups II, III, IV, V and VII in food outbreaks have been reported (Cadel Six et al., 2012). *B. cereus* is associated to a large number of food products such as rice, pasta and milk or mayonnaise-based ready-to-eat (RTE) food salad (Mortimer and McCann, 1974; Parry and Gilbert, 1980; Larsen and Jørgensen, 1999; Reyes et al., 2007; Valero et al., 2007; Delbrassinne et al., 2011). Nevertheless, the prevalence of *B. cereus *inducing food-borne deseases is difficult to determine and may be underestimated, because the symptoms associated with *B. cereus* infections or intoxications are generally mild and not always reported (Stenfors Arnesen et al., 2007; Cadel Six et al., 2012). However, more severe cases which may lead to fatal cases have also been reported, demonstrating the possible severity of the emetic syndrome (Dierick et al., 2005; Shiota et al., 2010; Naranjo et al., 2011).

*B. cereus* is also known for its ability to cause food spoilage which may lead to enormous expenses for food industry (Gram et al., 2002), mainly spoilage of milk and dairy products (Meer et al., 1991; Andersson et al., 1995; De Jonghe et al., 2010), thereby shortening the shelf-life of these products. Spoilage of pasteurized milk by *Bacilli* may cause defects such as off flavors and structural defects mainly due to the production of hydrolytic extracellular enzymes such as proteolytic, lipolytic and/or phospholipolytic enzymes (Meer et al., 1991). The off-flavors can be bitter, putrid, rancid, fruity, yeasty, or sour. Bitter flavor is caused by protease acitivity on the milk proteins, while rancid and fruity flavors are caused by lipases (Heyndrickx and Scheldeman, 2002). For instance, *B. cereus* produces a chymosin-like protease enzyme which is reportedly responsible for degradation of milk casein, resulting in coagulation (sweet curdling) and finally a bitter-tasting product (Frank, 1997). The production of a phospholipase C which degrades fat globule membranes, resulting in fat aggregation in cream, has been also reported in *B. cereus *(Frank, 1997). To prevent growth of microorganisms, food is often processed and preserved. Usually food products were sterilized by heat, but nowadays in response to consumers demand the food industry use milder conditions for preservation (Abee and Wouters, 1999). Combinations and applications of differents preservation steps, named hurdles, are frequently applied in modern food industries to ensure the microbial safety and stability and preserve the sensory and nutritional quality (Leistner and Gorris, 1995; Leistner, 2000). Mild heat treatment, low pH and cold storage are examples of hurdles used to preserve food. In order to cope with these environmental dynamics, microorganisms have developed stress adaptation strategies that lead to the possibility that bacteria overcome harsher conditions for a variety of stresses. The heat and salt stress responses of *B. cereus* have been studied (see for *e.g., *Browne and Dowds, 2001; Periago et al., 2002; van Schaik et al., 2004a; den Besten et al., 2007, 2009) for few years and more recently, the acid stress response of *B. cereus *has been investigated too (Mols et al., 2010a,b). In 2011, a minireview describing the acid stress response of both germinating spores and vegetative cells of *B. cereus* was published (Mols and Abee, 2011b). Both spores and vegetative cells of *B. cereus* could be found in food and in order to inactivate spores, drastic treatments are necessary but may affect food organoleptic properties. Thus, another way to limit the prevalence of *B. cereus* in food, could be to focus on vegetative cells. Indeed, in minimally processed food, initial contaminant spores may germinate and thus, inactivating or limiting the growth of the subsequent vegetative cells with mild preservation processes will allow to minimize the prevalence of *B. cereus* in such kind of food products.

*B. cereus* exhibits many differences with the Gram-positive model *B. subtilis*, such as the regulon of the general stress response (Anderson et al., 2005; de Been et al., 2011): this review will detail the vegetative cells acid stress response of* B. cereus *while data on the response of other Gram-positive bacteria upon exposure to low pH have been reviewed by Cotter and Hill (2003).

Here, mechanisms of acid resistance in *B. cereus* are reviewed as (i) the involvment of the general stress response in acid stress response, (ii) the pH homeostasis maintaining, (iii) metabolic rearrangements and alkali production, and (iv) the secondary oxidative stress response observed upon exposure to low pH.

## GENERAL STRESS RESPONSE

The ability of bacteria to respond rapidly to changing environmental conditions is a prerequisite for survival in their habitats. This bacterial stress response is triggered by a change in the microorganism growth conditions. Such a change triggers a cascade of events that will lead to increase stress resistance of the bacterial cell, most often against not only the stress to which it was exposed but also other stresses, thereby ensuring its survival under a variety of conditions. A common strategy that bacteria use to counter stressful conditions is to activate a specific alternative sigma factor, which leads to the transcription of a set of genes (a so-called regulon), the products of which protect the cell against adverse conditions (Kazmierczak et al., 2005). In several Gram-positive bacteria, the alternative sigma factor σ^B^ is the key sigma factor controlling the general stress response (van Schaik et al., 2004b; Kazmierczak et al., 2005). This factor is a secondary subunit of RNA polymerase that is known to play an important role in regulating gene expression when major changes in the environment occur. Upon binding σ^B^ to core of RNA polymerase, genes located downstream a promoter that can be recognized by the σ^B^-RNA polymerase complex are transcribed. The role of σ^B^ and its regulation has been extensively studied in the Gram-positive model-organism *B. subtilis* (for a comprehensive review, see Hecker et al., 2007). In *B. cereus*, σ^B^ is activated in several stress conditions such as ethanol, NaCl exposure or H_2_O_2_ and acid shock. However, the largest up-regulation of σ^B^ is observed in response to heat shock (van Schaik et al., 2004a).

### σ^B^ REGULATION

In all Gram-positive bacteria, the activation of σ^B^ confers protection to cell against adverse conditions. In *B. subtilis*, σ^B^ activity is controlled by RsbVW partner-switching, a mechanism which is highly conserved in species that contain σ^B^, including *B. cereus *strains (de Been et al., 2010, 2011). In non-stressed cells, σ^B^ is present in an inactive form by complexation with the anti-sigma factor RsbW. In this form, σ^B^ is unable to bind to RNA polymerase and thus cannot initiate the transcription of stress response genes. Under stress, an anti-sigma factor antagonist, RsbV, can bind to RsbW, thereby forming an RsbV-RsbW complex. This leads to the release of σ^B^, which can then bind to RNA polymerase, leading to the transcription of σ^B^-dependent genes. In addition, RsbW acts as a kinase of RsbV, thereby providing a negative feedback on σ^B^ activation. Under stress conditions, RsbV is dephosphorylated by one or more specific PP2C-type phosphatases, resulting in the sequestration of RsbW and the activation of σ^B^. This part of this regulatory is conserved in *Bacilli *(van Schaik et al., 2005), *Staphylococcus aureus *(Palma and Cheung, 2001; Senn et al., 2005; Pané-Farré et al., 2006) and *L. monocytogenes* (Wiedmann et al., 1998; Ferreira et al., 2004). However, there are considerable differences in the upstream part of the σ^B^ activation pathway (Ferreira et al., 2004; van Schaik et al., 2004a), reflecting differences in the mechanisms of stress sensing and signaling in the various bacteria.

**FIGURE 1:**
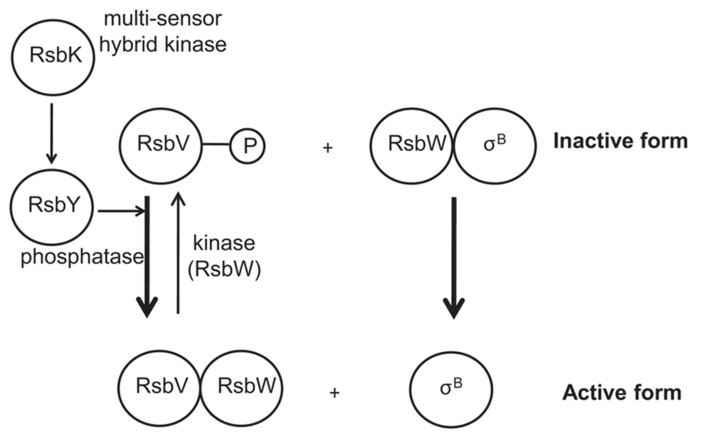
**Schematical representation of the activation of *Bacillus cereus *σ^B^.** Upon different stressing conditions, RsbK auto-phosphorylates a conserved histidine residue. The phosphoryl group is then transferred to RsbY. RsbV is then dephosphorylated by RsbY, resulting in the sequestration of RsbW and the activation of σ^B^. The anti-sigma factor RsbW also acts as a kinase of RsbV, thereby providing a negative feedback on σ^B^ activation.

**FIGURE 2:**
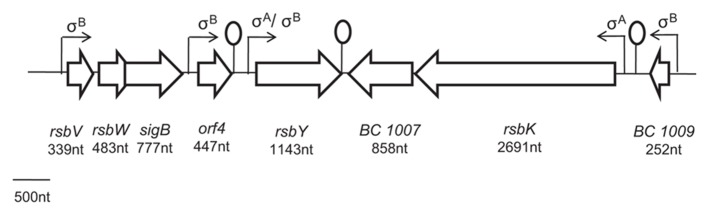
**Overview of the genetic organization of *sigB* cluster of *Bacillus cereus *ATCC 14579.** *sigB* code for the alternative sigma factor σ^B^and *rsbV, rsbW* and *rsbY *code for its regulatory proteins. *orf4* encode a bacterioferritin. *rsbK *encode a hybride kinase which may constitute with RsbY one functional module for the control of σ^B^ activity in members of the *B. cereus *group (de Been et al., 2011).

In the human pathogen *B. cereus*, the mechanism of σ^B^ activation has only been studied more recently (van Schaik et al., 2004a,b, 2005, 2007; de Been et al., 2010, 2011). It has been shown that σ^B^ activation is governed by a single PP2C-type phosphatase, RsbY, which carries an N-terminal response receiver (REC) domain (van Schaik et al., 2005). The *rsbY* gene is unique to *B. cereus* and to closest relatives and is transcribed both from σ^A^-dependent promoter (constitutive sigma factor) and σ^B^-dependent promoter. Because, the RsbY carries an N-terminal REC domain it could be suggested that RsbY is activated through a mechanism which involves phosphorylation of a conserved aspartate residue in the REC domain by a kinase. de Been et al. (2010) identified that this PP2C-type phosphatase RsbY receives its input from the multi-sensor hybrid kinase RsbK (**Figure 1**). RsbK contains both sensor histidine kinase and response regulator domains, and *rsbK* gene is located close to *sigB* on the genome of *B. cereus *ATCC 14579 (**Figure 2**). They also realized a genome survey (de Been et al., 2011) indicating that RsbK and RsbY should constitute one functional module for the control of σ^B^ activity in members of the *B. cereus *group, including the pathogens *B. thuringiensis*, *B. anthracis* and the psychrotolerant *B. weihenstephanensis.* One exception concerns the *B. cytotoxicus* genome which lacks the entire SigB operon, including *sigB* gene and the primary regulatory loci that control the SigB activity, *rsbV* and *rsbW *(Lapidus et al., 2008; Scott and Dyer, 2012). Orthologous RsbKY signaling modules were found in four other *Bacilli *outside the *B. cereus* group. However, the RsbKY modules in these other *Bacilli *strains were not connected to *sigB* in terms of genomic context (de Been et al., 2010). Analysis of the transcriptional organization of the σ^B^operon revealed that this operon is transcribed as a 2.1-kb transcript encompassing *rsbV, rsbW, sigB, *and *orf4* (**Figure 2**). o*rf4*, encoding a bacterioferritin (Wang et al., 2009), is also under the control of an additional σ^B^-dependent promoter and is a member of the σ^B^ regulon in *B. cereus. *The *rsbY* gene was found directly downstream of the σ^B^ operon in *B. cereus* group and is transcribed both from σ^B^ and σ^A^-RNA polymerase (van Schaik et al., 2004a; Wang et al., 2009). Once stress conditions is sensed and signaled through the regulatory cascade, the activation of σ^B^ as well as the transcription of the set of σ^B^-regulated genes occurs allowing bacterial resistance. van Schaik et al. (2004a) demonstrated that upon stress conditions, the level of σ^B^ raised rapidly. Thereby they showed that an addition of 4% ethanol, 2.5% NaCl as well as heat (42°C) or acid shock (pH 5.2) have an impact on σ^B^ level. They also highlighted a limited effect of ATP depletion on σ^B^ level, showing that, unlike in *B. subtilis*, the σ^B^ response could occur solely in response to changes of environmental conditions.

### σ^B^ REGULATED GENES AND ACID STRESS

As mentioned, the activation of the σ^B^ response will allow the transcription of the set of genes coding for proteins with specific functions, which will protect the cell against stress. The identification of the complete σ^B^ regulon in *B. subtilis* (Helmann et al., 2001; Petersohn et al., 2001; Price et al., 2001), *L. monocytogenes* (Kazmierczak et al., 2003), and *Staphylococcus aureus *(Bischoff et al., 2004; Pané-Farré et al., 2006) by DNA microarray technology revealed that a low number of genes have an obvious role in the stress response of these organisms and may contribute to stress resistance. For instance, *L. monocytogenes* σ^B^ regulates the *gadB *gene (Kazmierczak et al., 2003) that is involved in acid stress resistance (discuss in the see Amino-Acids Decarboxylase Systems) and into several metabolic pathways like the amino-acids pathway. Furthermore, in both L. monocytogenes and S. aureus, some pathogenic traits seem to be governed by σ^B^ through the control of the expression of virulence gene regulators (Bischoff et al., 2004; Kazmierczak et al., 2005). While in *B. cereus* ATCC 14579 and in a *sigB* null mutant, the production of virulence factors is the same (van Schaik et al., 2004a), a *B. anthracis *Δ*sigB* strain was affected in its virulence (Fouet et al., 2000). Indeed, the injection of spores of *B. anthracis* 7702 strain or its Δ*sigB* derivative in 10 mice showed that the number of deaths with given doses of the Δ*sigB *strain were similar to those obtained with the 1 log unit lower doses of the parental strain suggesting a 1 log unit difference in the 50% lethal dose (Fouet et al., 2000). These findings suggest that σ^B^ has evolved in response to specific niches (Ferreira et al., 2004). In addition, the *B. cereus* σ^B^ regulon differs from *B. subtilis, L. monocytogenes* and* Staphylococcus aureus *ones. Actually, it has been highlighted that the transcription of approximately 30 genes are σ^B^-dependent in *B. cereus* ATCC 14579 (van Schaik et al., 2004a,b, 2007; de Been et al., 2010) among which the majority are organized in operons (**Table 1**) whereas around 100 genes are σ^B^-dependent in *B. subtilis *and *Staphylococcus aureus* (Helmann et al., 2001; Petersohn et al., 2001; Bischoff et al., 2004; Pané-Farré et al., 2006). In *L. monocytogenes*, 54 genes were up-regulated in a σ^B^-dependent fashion upon stress exposure. However, the total number of σ^B^-dependent genes in *L. monocytogenes* may be higher, as the microarray used in that study had only limited genome coverage (Kazmierczak et al., 2003).

**Table 1:** *Bacillus cereus *ATCC 14579 σ^B^ regulon members adapted from de Been et al. (2010).

Gene accession	Alias	Annotation
BC0861		Hypothetical protein
BC0862	*yfkM*	Protease I
BC0863	*katE*	Catalase
BC0995		Hypothetical protein
BC0996		Hypothetical protein
BC0998	*yflT*	General stress protein
BC0999	*csbD*	Hypothetical protein
BC1000		Hypothetical protein
BC1001		Hypothetical protein
BC1002	*rsbV*	Anti-σ^B^ factor antagonist
BC1003	*rsbW*	Anti-σ^B^ factor
BC1004	*sigB*	RNA polymerase sigma factor σ^B^
BC1005	*orf4*	Putative bacterioferritin
BC1006	*rsbY*	PP2C-type RR; regulates σ^B^activity
BC1007	*cheR*	Chemotaxis protein methyltransferase
BC1008	*rsbK*	Multi-sensor hybrid kinase
BC1009		Hypothetical protein
BC1010		Hypothetical protein
BC1011		Hypothetical protein
BC1012	*ybjQ*	Hypothetical protein
BC1154	*hemH-2*	Ferrochelatase
BC1155	*katA*	Catalase
BC2108	*sigZ*	ECF sigma factor
BC2638		Spore germination protein LC
BC3129	*corA*	Mg^2+^ and Co^2+^ transporter
BC3130		Hypothetical protein
BC3131		Hypothetical protein
BC3132	*yflT*	General stress protein
BC4640	*ytfJ*	Hypothetical protein
BC4641	*ytfI*	Hypothetical protein
BC5390	*cwIJ*	Cell wall hydrolase
BC5391	*gerQ*	Spore coat protein

The comparison of *B. cereus, B. subtilis, L. monocytogene*s and *Staphylococcus aureus* σ^B^ regulons showed that genes are generally conserved among the four Gram-positive bacteria to same extent as all the other genes of the genome. However, only three genes (*rsbV, rsbW, *and *sigB*) are conserved in their σ^B^ dependency in all four bacteria, suggesting that the σ^B^ regulon of the different Gram positive bacteria has evolved to perform niche specific functions (van Schaik et al., 2007). Composed of 14 unknown proteins, the role of the σ^B^ regulon of *B. cereus* to counteract the acid stress has not been studied in detail. Microarray analyses were performed on *B. cereus *ATCC 14579 cells exposed 10 min to a lethal (pH 4.5) or sublethal (pH 5.4) acid stress (Mols et al., 2010b): the variation of the σ^B^-dependent gene expression was reported except for BC0999 and BC1012 genes coding for two hypothetical proteins. Eleven genes belonging to the σ^B^ regulon showed an over-expression both in lethal and non-lethal acid stress. Among them, the σ^B^ operon containing *rsbV, rsbW* and *sigB, orf4*, and *rsbY*, as well as the two genes *BC1000 *and *BC1009* are up-regulated (≥1.5 fold in both conditions). In the same way, *BC0862 *and *BC0998* genes are over-expressed: the first one encoding the YflT protein is known to be heat-shock inducible both in *B. cereus *and *B. subtilis* but its role in stress response remains unclear, whereas the second gene encodes the YfkM protease that may acts to degrade incorrectly folded proteins (Helmann et al., 2001; van Schaik et al., 2004b). The genes *BC1154 *encoding a ferrochelatase and *BC1155* encoding the main vegetative catalase KatA were up-regulated both in lethal and non-lethal conditions but more upon mild acid exposure (from 7.8 to 44 fold) than upon lethal acid stress (from 1.5 to 3.7 fold). Eight other σ^B^-dependent genes confirms that the response differ with the intensity of acid stress: for example, *BC0863* and* BC3129* genes, encoding respectively the catalase KatE and a magnesium cobalt transporter CorA, were up-regulated upon non-lethal acid stress whereas they were down regulated in lethal acid stress.

Furthermore, *clp, groES, *and* dnaK* genes encoding repair and chaperone proteins and heat stress regulators *ctsR* and *hrcA* (van de Guchte et al., 2002) were also shown to be up-regulated upon exposure to acid conditions in *B. cereus *(Mols et al., 2010b; Mols and Abee, 2011b).

## pH HOMEOSTASIS

The external pH as well as the presence of weak acid determines the intracellular pH (pH_int_). Weak acids under their unprotonated form can diffuse into the cell and dissociate, releasing a proton and leading to acidification of the cytoplasm. Strong acids are not able to permeate through the cell membrane but by lowering the external pH and increasing the pH gradient, they increase the proton permeability and thereby lead to the reduction of pH_int_ (Krebs et al., 1983; Abee and Wouters, 1999; Cotter et al., 2000; Beales, 2004). Bacteria can survive thanks to their ability to regulate their pH_int_, a process primarily driven by controlled movement of cations across the membrane (Beales, 2004). However, this ability to maintain pH_int_ (pH homeostasis) can be overtaken at low extracellular pH value, leading to the cell death (Booth and Kroll, 1989; Beales, 2004). For instance, *B. cereus* ATCC 14579 cells grown at pH 7.0 or 5.5 showed pH_int_ values of 7.10 and 6.22, respectively, whereas a 40 min exposure of cells at pH 4.0 decreased the pH_int_ of 2.02 units and was combined to a population reduction of 2.35 log (Senouci-Rezkallah et al., 2011).

Therefore to survive in acidic conditions, many microorganisms activate enzymes contributing to maintain their pH homeostasis (Mols and Abee, 2011b), such as proton pumps and consuming proton reactions like glutamate decarboxylation.

### F_1_F_0_-ATPase AND PROTON TRANSPORTER

In aerobic organisms, the active transport of H^+^ is coupled with electron transport in respiratory chains whereas anaerobic bacteria carry out H^+^ transport via H^+^-ATPase molecules using energy from ATP hydrolysis (Shabala et al., 2002; Gandhi and Chikindas, 2007). In *L. monocytogenes, *a facultative anaerobic bacterium, the use of both processes has been demonstrated (Shabala et al., 2002). The multi-subunit enzyme F_1_F_0_-ATPase, that is highly conserved, serves as a channel for proton translocation coupled with ATP synthesis or hydrolysis (Fillingame and Divall, 1999; Yoshida et al., 2001). ATP synthesis is generated by the expense of proton motive force (PMF) whereas ATP hydrolysis generates a PMF that can facilitate the extrusion of proton from the cytoplasm (Cotter and Hill, 2003).

Since *B. cereus *is a facultative anaerobic bacterium, it could be supposed that this bacterium may use both ATP hydrolysis and synthesis to maintain its pH homeostasis as shown in *L. monocytogenes*. In fact, F_0_F_1_-ATPase encoding genes were down-regulated in *B. cereus *ATCC 14579 and* B. cereus *ATCC10987 exposed to non-lethal acid conditions, and were not repressed upon exposure to lethal acid stresses, indicating that *B. cereus* does not use F_0_F_1_-ATPase to extrude proton in aerobic conditions (Mols et al., 2010a,b). Down regulation of F_0_F_1_-ATPase genes could be explained by the cells trying to prevent excessive inward flux of proton via this ATPase upon exposure to acid conditions (Mols et al., 2010b). This down-regulation has also been demonstrated in *Staphylococcus aureus* where the expression of F_0_F_1_-ATPase encoding-genes was clearly reduced to about 50% (Bore et al., 2007). But in these cases, the ATPase could still play an important role by pumping H+ out of the cells, since Arikado et al. (1999) suggest that the enzyme regulation occurs mainly at the post-transcriptional level. Indeed, they showed that regulation of the enzyme level of F1F0-ATPase by the intracellular pH, in Streptococcus faecalis, takes place especially at the step of enzyme assembly from its subunits.

Other proton transporters may also play key role in pH homeostasis: indeed, in early 1970s, it has been shown that an activity in biological membranes couples the fluxes of Na^+^ and H^+^ (Mitchell, 1961; Mitchell and Moyle, 1967; West and Mitchell, 1974). They suggested that Na^+^/H^+^ antiporters are involved in the homeostasis of both Na^+^ and H^+^ in cells. Since then, Na^+^/H^+^ as well as K^+^/H^+^ antiporter activity has been found in cytoplasmic membranes of many cells. These monovalent cation/proton antiporters are especially known to be involved in bacterial pH homeostasis under alkaline challenge (Krulwich et al., 1999). Interestingly, Mols et al. (2010b) showed that napA encoding a Na+/H+ antiporter was highly up-regulated in B. cereus cells exposed to lethal pH conditions whereas it was down regulated upon non-lethal acid exposure (Mols et al., 2010b).

Thus, F0F1-ATPase and antiporters gene regulations under lethal and non-lethal conditions in B. cereus cells indicated a fine balance between ATP synthesis on one hand and proton pumps regulating pHint at the expense of ATP on the other hand (Mols and Abee, 2011b).

### AMINO-ACIDS DECARBOXYLASE SYSTEMS

Amino acid decarboxylases function to control the pH of the bacterial environment by consuming hydrogen ions as part of the decarboxylation reaction (Gale and Epps, 1942; Cotter and Hill, 2003; Beales, 2004; Bearson et al., 1997). Examples of this are the lysine, arginine, and glutamate decarboxylases (GADs), which operate by combining an internalized amino acid with a proton and exchanging the resultant product for another amino acid substrate. Furthermore, the products of amino acid decarboxylases consist of basic amines (pKa value around 10) which may be responsible of a slight increase of extracellular pH (Olson, 1993). Lysine decarboxylase is an enzyme that converts lysine to cadaverine and was found to be an important system involved in acid resistance and in pH_int_ homeostasis in several Gram-negative bacteria including *E. coli, Vibrio vulnificus, Vibrio parahaemolyticus, *and* Salmonella typhimurium* (Bearson et al., 1997; Eun Rhee et al., 2002; Moreau, 2007; Tanaka et al., 2008). Arginine decarboxylase enzyme has one substrate (L-arginine) and two products, CO2 and agmatine, which has been showed to be a competitive inhibitor in *E. coli* (Blethen et al., 1968). GAD converts glutamate in γ -aminobutyrate (GABA) and is well described in *L. monocytogenes* for instance (Cotter et al., 2001; Gandhi and Chikindas, 2007; Karatzas et al., 2010, 2012; Feehily et al., 2013).

**FIGURE 3:**
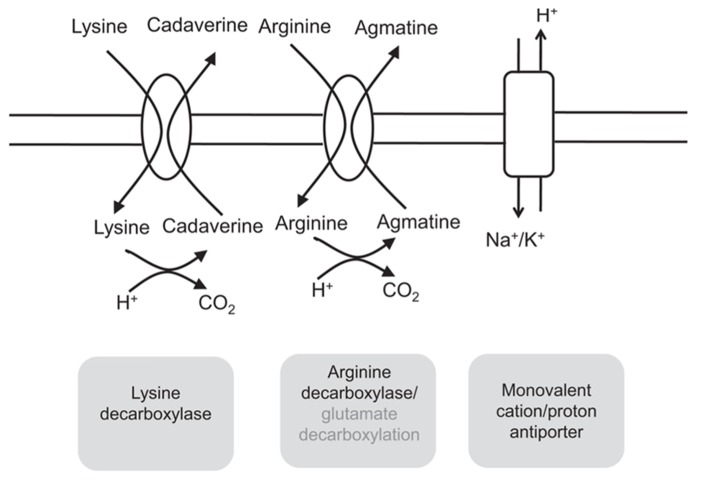
**Graphical representation of the biological systems involved in pH homeostasis of *Bacillus cereus *cells.** Lysine decarboxylase converts lysine to cadaverine, arginine decarboxylase converts arginine to agmatine, consuming a proton and producing a CO_2_. Glutamate decarboxylation converts glutamate in γ-aminobutyrate, Note that this enzyme is not present in all *B. cereus* strain as shown for *B. cereus* ATCC 14579 and in this strain the glutamate may be decarboxylated by the arginine decarboxylase. Monovalent cation/proton antiporters may also play key role, by proton extrusion, in pH homeostasis of *B. cereus* particularly upon exposure to lethal pH.

Today, only few pieces of information are available on these systems in *B. cereus*. Mols et al. (2010b) showed that the glutamate decarboxylase gene (*gad*) is not present in the genome of *B. cereus* ATCC 14579 whereas it is present in *B. cereus *ATCC 10987 and many other strains. However, they did not see any difference in growth under acidic conditions between these strains. As previously described for *L. monocytogenes *(Cotter et al., 2001), it may be possible that the GAD does not contribute to the growth of *B. cereus* ATCC 10987 in acidic environments because the gene encoding the glutamate/GABA antiporter is lacking. Senouci-Rezkallah et al. (2011) studied the impact of amino-acid presence on the acid tolerance response of *B. cereus *ATCC 14579 strain. They observed that survival to an acid shock at pH 4.0 of *B. cereus* cells grown at pH 7.0 was enhanced in the presence of glutamate and strongly enhanced in the presence of arginine or lysine. However, the presence of these amino-acids had no impact on the acid tolerance of acid pre-adapted cells when submitted to acid shock. They also observed that the presence of glutamate, arginine or lysine increased the pH_int_ of *B. cereus* cells grown at pH 7.0 during exposure at pH 4.0 whereas it had no significant influence on pH_int _cells grown at pH 5.5 during acid shock, suggesting an induction of these systems under these growth conditions (**Figure 3**). As *B. cereus* ATCC 14579 does not possess the gene encoding for the GAD, the glutamate may be decarboxylated by the arginine decarboxylase, as described in *Lathyrus sativus* (Ramakrishna and Adiga, 1975).

## METABOLISM MODIFICATION AND ALKALI PRODUCTION

Depending on their environment, bacteria will establish specific pathways allowing their survival or growth. Several metabolic pathways have been associated with bacterial growth at low pHs. Upon acid stress, alkali production is often observed and well established in oral bacteria such as *Streptococcus gordonii, Streptococcus parasanguis, Streptococcus rattus, Streptococcus sanguis *(Burne and Marquis, 2000). Urea and arginine are two major substrates for alkali generation by oral biofilms colonizing the teeth (Lemos and Burne, 2008). Urea is in all salivary gland secretions and is rapidly hydrolyzed to ammonia and CO_2_ by bacterial ureases while arginine is primarily catabolised to ornithine, ammonia and CO_2_. The increased tolerance of cells results from the production of NH_3_, which combines with protons in the cytoplasm to produce ${NH}_{4}^{+}$ raising the internal pH. Nevertheless, it could bear in mind that these two systems are not present in all oral bacteria (Lemos and Burne, 2008) and other mechanisms play important role in their acid tolerance.

### AMMONIA PRODUCING MECHANISM

**FIGURE 4:**
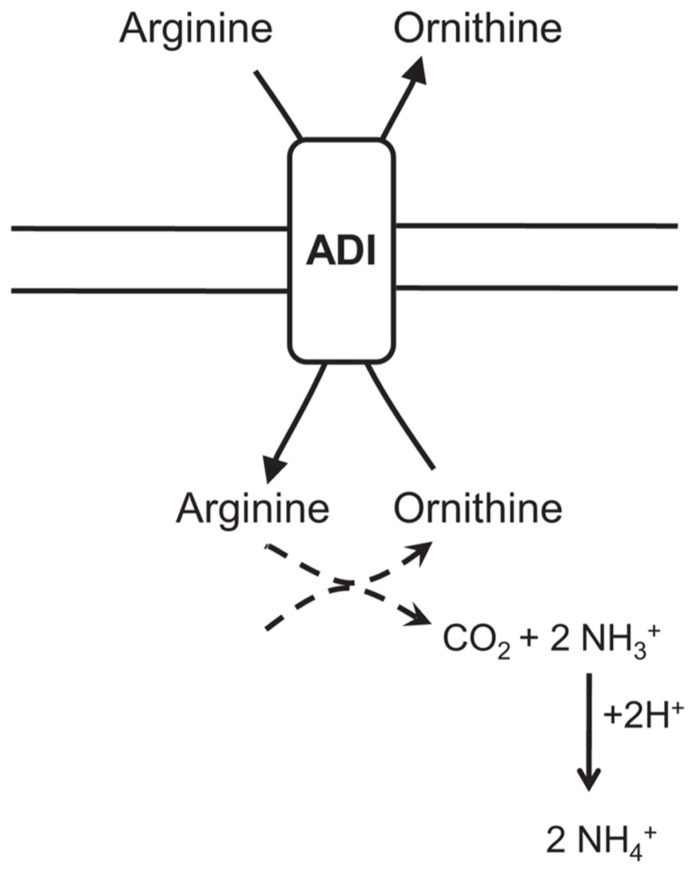
**Graphical representation of the arginine deiminase (ADI) pathway implied in the acid stress response of *Bacillus cereus*.** The ADI pathway converts arginine to citrulline and ammonia. Citrulline is then metabolized into ornithine generating carbon dioxide and ammonia. Arginine and ornithine are exchanged via an antiporter importing arginine and exporting ornithine. Ammonia reacts with proton forming ammonium.

Arginine deiminase or the ADI pathway (for review, see Lu, 2006) has been identified in a variety of Gram-positive bacteria, including *Bacillus *spp., *L. monocytogenes*, and several lactic acid bacteria (LAB; Cunin et al., 1986; Ryan et al., 2009). The ADI pathway converts arginine to citrulline and ammonia. Subsequently, citrulline is metabolized into ornithine generating carbon dioxide, ammonia and ATP. Arginine and ornithine are exchanged via an antiporter importing arginine and exporting ornithine. The resulting NH_3_ rapidly reacts with H^+^ and helps to alkalize the environment consuming proton and forming ammonium. Furthermore, the generated ATP can enable extrusion of cytoplasmic protons by the F_0_F_1_-ATPase as shown in *L. monocytogenes* or in LAB (van de Guchte et al., 2002). This system has three main enzymes, ADI, ornithine transcarbamylase, and carbamate kinase, encoded by *arcA, arcB* and *arcC*, respectively. They appear to be inherently acid-tolerant, displaying activity at pH 3.1 and even lower in some species (Casiano-Colón and Marquis, 1988; Curran et al., 1995). The role of the ADI pathway in acid stress resistance has been established in bacteria such as *streptococci* and *L. monocytogenes*: *S. sangui* showed ADI activity upon exposure to pH 3.5 leading to an increase resistance (Curran et al., 1995) and ADI negative mutants of *L. monocytogenes* were more sensitive to low pHs (Ryan et al., 2009). In *B. cereus*, arginine deiminase gene *arcA* showed significant up-regulation in *B. cereus* ATCC 14579 and ATCC 10987 upon exposure to non-lethal acid shock at pH 5.4, whereas exposure to a lethal acid shock pH 4.5 revealed no significant induction (Mols et al., 2010b). Senouci-Rezkallah et al. (2011) also demonstrated a six-fold up-regulation of the *arcA* gene in acid adapted cells grown at pH 5.5 compared to non-preadapted cells grown at pH 7.0. These data suggest that the ADI pathway may be of great importance for *B. cereus *survival in low pH environments. Nevertheless, this role seems to be restricted to non-lethal acid stress (**Figure 4**).

Another ammonia producing mechanism associated with low pH resistance is urease. The urease enzyme catalyzes the hydrolysis of urea, generating two molecules of ammonia and one molecule of carbon dioxide. Urease is well studied in the human pathogen *Helicobacter pylori *in which urease plays an important role in the ability of stomach colonization and in virulence (Eaton et al., 1991). Urea is present in various environments in which *B. cereus* can be found, including soil, food, and the human body, where urea is present in all fluids and is finally excreted in the urine as a detoxification product (Mobley and Hausinger, 1989; Burne and Chen, 2000). Rasko et al. (2004) identified an urease cluster composed of nine genes (from *BCE3657* to *BCE3666*) in the genome of *B. cereus *strain ATCC 10987 that is not present in all sequenced strains belonging to *B. cereus* (Mols and Abee, 2008). This cluster harbors three genes *ureA, ureB* and *ureC*, encoding structural enzymes, four genes (*ureEFGD*) encoding accessory proteins, and two additional genes *ureI* and *nikT* respectively encoding a putative urea (acetamine) transporter and a nickel transporter. Mols and Abee (2008) showed that among 49 *B. cereus* strains, isolated from various sources and including clinical, environmental and food isolates, together with the type strain ATCC 14579 and other sequenced strains such as ATCC 10987 and PAL25, 10 strains are *ureABC* PCR-positive and nine also possess urease activity. They also showed that urease activity did not contribute to *B. cereus* survival capacity under acid shock conditions.

### FERMENTATIVE PATHWAYS

The metabolic pathways involved in fermentative growth, such as lactate, alcohol and butanediol pathways are involved in *B. subtilis* cell acid stress response as shown by their up-regulation upon low pH of growth (Cruz Ramos et al., 2000; Wilks et al., 2009). AlsS condenses two molecules of pyruvate to form acetolactate, converted to acetoin by spontaneous decarboxylation at low pH and by the action of the acetolactate decarboxylase (AlsD; Renna et al., 1993). Acetoin can then be excreted by the cells. Alcohol dehydrogenase (Adh) is an NAD(P)-dependent dehydrogenase that may remove acidity and transfer electrons to the electron transfer chain (ETC; Reid and Fewson, 1994). Lactate dehydrogenase, encoded by *ldh*, is a cytoplasmic NADH-linked enzyme that converts pyruvate to lactate to remove acidic compounds, restoring the NAD^+^/NADH balance. During fermentation, Ldh is the key enzyme involved in reoxidation of the NADH formed by glycolysis (Cruz Ramos et al., 2000). In *Streptococcus oralis* and *Streptococcus mutans*, it was shown that many enzymes involved in glycolysis are up-regulated during growth at low pH (Wilkins et al., 2001, 2002). It has been suggested that the increase in the amount of these proteins may result in an increase in ATP production and consequently increased proton extrusion via the F_1_F_0_-ATPase. In *B. subtilis*, acid conditions also up-regulate a large number of NAD(P)-dependent dehydrogenases such as alanine dehydrogenase (*ald*), succinate-semialdehyde dehydrogenase (*gabD*), and several putative formate dehydrogenases (*fdhD, yjgC, yrhE, and yrhG*). These enzymes are able to remove acidity through NAD(P)H which transfers electrons to the electron transport system and pumps protons out of the cell (Wilks et al., 2009).

Mols et al. (2010a) showed that a set of 25 genes of *B. cereus *ATCC 14579 are differentially expressed in lethal or non-lethal organic or inorganic acid conditions and a set of 146 genes for all non-lethal acid conditions. Up-regulation concerned mainly genes involved in energy metabolism, oxidative and general stress response. Pyruvate metabolism, the tricarboxylic acid cycle (TCA) and fermentation pathways were induced to maintain intracellular ATP levels and/or the redox balance. In lethal acid conditions, increase of lactate dehydrogenase (*ldh*) and cytochrome bd oxidase (*cydAB*) gene expression are shown. In *B. subtilis*, these genes are co-ordinately expressed together with the lactate permease gene *lctP* and the formate-nitrite transporter gene *ywcJ *and under control of the negative regulator YdiH (Rex; Larsson et al., 2005). Lactate dehydrogenase in concert with the cytochrome bd oxidase has been proposed to function as an alternative electron transport chain (Chai et al., 2009). The *alsSD* is also up-regulated upon exposure to lethal or non-lethal acid stresses, but this up-regulation is less pronounced upon lethal conditions than under non-lethal acid stress. Together with the *alsSD* genes, *cydAB*, *ldh* and *lctP* form a distinct regulon, which is part of the Fnr regulon (Reents et al., 2006). By analogy with *B. subtilis*, the induction of these genes may be associated to a changing in NADH/NAD^+^ ratio. It could be noted that under lethal concentration of acetic acid *B. cereus* showed some resemblance with the response of *Staphylococcus aureus *cells deficit of *murF* which exhibit a reduced peptidoglycan synthesis. Indeed, these cells down-regulated iron uptake associated genes and, induced *ldh*, lactate permease, and formate/nitrite transporter protein genes (Sobral et al., 2007).

**FIGURE 5:**
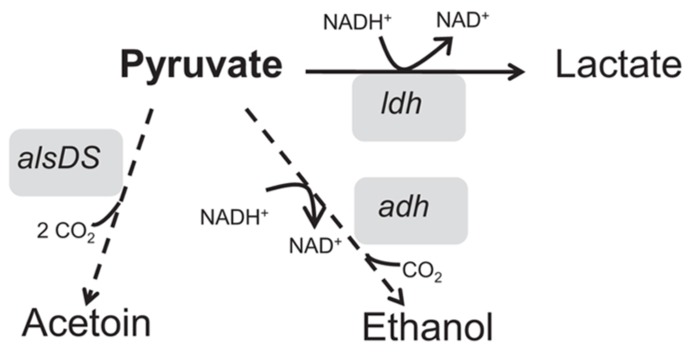
**Graphical representation of the fermentative pathways, such as acetoin production (*alsDS*), alcohol (*adh*) and lactate dehydrogenases (*ldh*), which represent the most notable metabolic rearrangements showed upon exposure to acid stress conditions**.

Mols et al. (2010b) also investigated the impact of lethal hydrochloric acid stress on *B. cereus *ATCC 14579 cells. Genes encoding for Adhs and lactate dehydrogenases appeared to be also induced upon exposure to lethal acid stresses (**Figure 5**). Therefore, the conversion of pyruvate to ethanol or lactate, generating CO_2_ and dissipating H^+^, may be an ultimate futile response of *B. cereus* to deal with low intracellular pH_int_ or restoration of NAD^+^/NADH balance. Furthermore, some metabolism rearrangements were also found specially correlated with lactic acid or acetic acid stress. For instance, several genes involved in glycolysis are moderately up-regulated upon exposure to lactic acid stress (2 mM undissociated acid; Mols et al., 2010a). Nevertheless, their functions remain to be elucidated.

## SECONDARY OXIDATIVE STRESS

The limited tolerance for oxygen is evident in the cases of obligate anaerobes and microaerophiles microorganisms, which cannot grow in air saturated media, but it applies as well to aerobes bacteria which also deal with the toxic side-effect of O_2_. Indeed, aerobic organism uses molecular oxygen (O_2_) for respiration or oxidation of nutrient to obtain energy. Reactive by products of oxygen, such as superoxide ($O_{2}^{-}$), hydrogen peroxide (H_2_O_2_), and the highly reactive hydroxyl radicals (OH^•^), are generated continuously in cells growing aerobically. Therefore, aerobic microorganisms could survive only because they contain antioxidant defenses. The biological targets for the reactive oxygen species (ROS) are DNA, RNA, proteins, and lipids. In some bacteria, such as *Borrelia burgdorferi, *the membrane could be the primary targets of ROS (Boylan et al., 2008). However, it has been shown that the most damaging effect of ROS in bacteria result from the interactions of H_2_O_2_ with Fe^2+^, generating reactive OH by the Fenton reaction. Because the Fe^2+^ is localized along the phosphodiester backbone of nucleic acid, DNA is a major target of OH (reviewed by Imlay, 2003). The effect of ROS on proteins is the oxidation of thiols, resulting in disulfide bond formation (Leichert et al., 2003). Living organisms have developed various defenses to protect themselves against ROS damage, some are enzymatic (catalases, superoxide dismustases (SOD), thioredoxins, and peroxidases) and others are non-enzymatic (glutathione, vitamin A, C). In bacteria, oxidative stress is sensed and lead to the activation of specific transcriptional regulators which will induce defense mechanisms when ROS concentration exceeds a critical level (Demple, 1991; Farr and Kogoma, 1991; Storz and Imlay, 1999). Furthermore, it is known that some other stresses could generate a bacterial response similar to the oxidative stress response. Indeed, this secondary oxidative stress response have been described upon exposure to salt, heat or cold temperatures, bile, starved and more recently upon lethal antibiotics conditions for *Bacillus *(Hecker and Völker, 2004; Hecker et al., 2007; den Besten et al., 2009; Ceragioli et al., 2010; Mols and Abee, 2011a).

Mols et al. (2010b, 2011a,b) showed that acid-stressed *B. cereus* cells revealed a major oxidative response, as other *Bacilli*. In sum, *B. cereus *cells exposed to lethal or non-lethal organic or inorganic acid stresses induce a set of genes such as those coding for superoxide dismutase, catalases and thioredoxins, which are known to be involved in bacterial oxidative stress response. Using fluorescent probes HFP, authors correlated the induction of these genes upon lethal conditions to the formation of ROS. Because the formation of ROS is apparent in *Bacillus *spp*.* upon lethal stresses (Hecker and Völker, 2004; Mols et al., 2009; Mols and Abee, 2011a), ROS may be part of a common mechanism of cellular death in respiring *Bacillus *species. Based on phenotypic and transcriptomic results, a model for environmental stresses was proposed with induction of radical formation, including OH^•^ and ONOO^-^ in *Bacillus *spp. (Mols and Abee, 2011a). Rapidly, environmental stresses induce perturbation of the ETC, where free electrons prematurely leak to oxygen resulting in the formation of superoxide ($O_{2}^{-}$). The ETC perturbation is confirmed by the expression of genes encoding alternative electron donor and acceptor mechanisms, such as cytochrome d ubiquinol oxidase (CydAB) and nitrate/nitrite reductase (Nar/Nas). Upon the action of superoxide dismutase, the $O_{2}^{-}$ is converted into hydrogen peroxide H_2_O_2_ leading to a primary oxidative stress response (for a critical review of *B. subtilis* oxidative stress response, see Zuber, 2009). By Fenton reaction and iron-sulfur cluster damaging, the H_2_O_2_ could be transformed into the highly toxic OH^•^ radicals. Furthermore, $O_{2}^{-}$ can rapidly react with nitric oxide (NO) to form another highly toxic compound, ONOO^-^, which may have a damaging effect that could lead to cell death (for more details, see Mols and Abee, 2011a).

## FROM PHYSIOLOGICAL KNOWLEDGE TO THE IDENTIFICATION OF POTENTIAL BIOMARKERS

The knowledge regarding the adaptive stress response of bacteria is of interest for the food industry, particularly with the increasing trend in the production of minimally processed foods where various stresses are combined to control microbial growth. Indeed, the implementation of the bacterial adaptive traits in predictive microbiology concept will lead to a more accurate prediction of the bacterial behavior under specific conditions. However, one of the major challenges will be to increment these data in decision making tools developed to support food safety and quality issues (McMeekin et al., 2008; Havelaar et al., 2010; Rantsiou et al., 2011; Brul et al., 2012). Up to now, two main different approaches to integrate bacterial behavior into predictive modeling are known with the identification and quantification of (i) signaling and metabolic pathway with flux balance analysis (Kauffman et al., 2003; Metris et al., 2012), or (ii) biomarkers (den Besten et al., 2010, 2013; Desriac et al., 2012, 2013). The official US National Institute of Health definition of a biomarker is “a characteristic that is objectively measured and evaluated as an indicator of normal biologic processes, pathogenic processes, or pharmacologic responses to a therapeutic intervention” (Atkinson et al., 2001). When applied to industrial process conditions, we propose to adapt this commonly medical oriented definition as: a characteristic that is objectively measured and evaluated as an indicator of bacterial responses to food processes and stress conditions. den Besten et al. (2010) described a strategy to identify biomarkers for cell robustness of *B. cereus*. Shortly, both unstressed and mild stress treated cells were exposed to lethal stress conditions (severe heat, acid and oxidative stress) to quantify the robustness advantage provided by mild stress pre-treatment. Robustness was defined by the ratio of log_10_N_(t__)_/log_10_N_0_, with N_(t)_ being the survivors quantified on agar plate at a defined time and N_0_ the inoculum (unstressed cells) used for inactivation treatment. This framework enabled the identification of candidate biomarkers, i.e., enzymatic activity, protein levels and genes expression obtained by reverse transcription followed by quantitative PCR (RT-qPCR) quantifications. Linear correlations between induced biomarker and induced robustness upon exposure to mild stress, revealed three kinds of biomarkers defined as “no-response biomarker,” “short-term biomarker” and “long-term biomarkers.” If this first approach proposed linear correlation, in 2012 we proposed an integrative approach encompassing both gene expression quantification throughout bacterial inactivation and mathematical modeling of the bacterial behavior to identify different molecular biomarkers to further predict the acid resistance of *B. weihenstephanensis*. The *sigB *gene was proposed as biomarker to track moderate acid resistance whereas* katA* was identified as biomarker to track high acid resistance. Indeed, fitting surviving bacterial counts of *B. weihenstephanensis* under lethal acid conditions (pH 4.6), allowed the identification of a biphasic patterns meaning that the bacterial population could be divided into two subpopulations with different acid resistances. By correlating the proportion of the two subpopulations with the gene expression, it could be shown that the highest expression of *sigB *was observed when the sensitive subpopulation represented the majority of the bacterial population. At the opposite the *katA* up regulation was correlated to the more resistant subpopulation. In 2013, we also proposed both linear and non-linear correlations between gene expression and acid survival ability of short adapted cells. Two kinds of biomarkers were defined with (i) direct biomarker genes for which the expression patterns upon mild stress treatment were linearly correlated to induced acid resistance; and (ii) long-acting biomarker genes which were up regulated for mild stress adaptation times of 30 min at maximum and linked to increased resistance over studied time (60 min).

However, if the selection of potential biomarkers offers new perspectives for the prediction of bacterial behavior and physiology, one of the key challenges will be to increment these data into mathematical model to predict growth or inactivation, during industrial processes to offer decision making tools for food safety and quality management (Brul et al., 2012; Desriac et al., 2012, 2013). The development of such models will contribute to support the food business sector competitiveness by optimizing inactivation process monitoring, eco efficient processing and accurate shelf life products. Thus, the integration of bacterial physiological state into predictive microbiology behavior will offer new tailor made decision making tools which could be used for getting the proper balance between food safety and food quality.

## CONCLUSION

Nowadays, food industries use mild preservation and processing techniques, in response to consumers demand for fresher, healthier and better foods and because mild preservation techniques save energy and are more environmental friendly. These mild preservation techniques, such as hurdles, may lead to the survival of spoilage and/or pathogenic microorganisms. Therefore the adaptive stress response and the physiology of bacteria is an important subject. *B. cereus* which can spoil food and cause food-borne illnesses encounter acid conditions in foods and upon ingestion and has to overcome the acid barrier of the human stomach (Clavel et al., 2004; Wijnands et al., 2009). For its control in food, it is especially relevant to understand how *B. cereus *cells grow and survive in acidic conditions.

**FIGURE 6:**
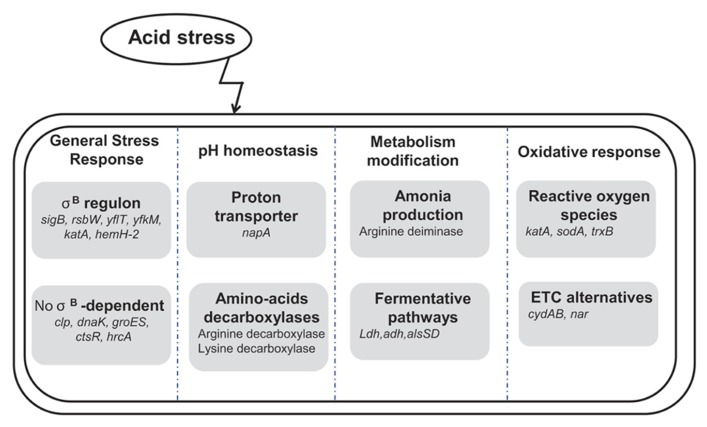
**Global representation of acid stress-associated mechanisms in *Bacillus cereus* which could be**divided into four different groups: (i) general stress response, (ii) pH homeostasis, (iii) metabolic rearrangements and (iv) oxidative response. The general stress response is governed by the σ^B^ factor (encoded by *sigB*) which regulated the expression of approximately 30 genes among which *rsbW* (anti σ^B^ factor), *yflT* (general stress protein), *yfkM* (protease), *katA* (catalase), *hemH-2* (ferrochelatase).** However, this group also contains genes which are putatively not only induced by low pH, but may be involved in a more general response to stresses and which are not under the control of σ^B^ such as *clp *(protease), *dnaK, groES* (chaperone proteins), *ctsR *and *hrcA *(transcriptional regulators). pH homeostasis involves monovalent cation/proton antiporter (*napA*) and amino-acids decarboxylases. The most notable metabolic rearrangements were fermentative pathways, such as acetoin production (*alsDS*), alcohol (*adh*) and lactate dehydrogenases (*ldh*) and the production of ammonia throughout the arginine deiminase system. At last, due to the perturbation of the electron transfer chain (ETC) upon acid stress exposure, an oxidative response involving catalase (*katA*), dismutase (*sodA*), thioredoxins such as *trxB* and alternative for the ETC such cytochrome *d* ubiquinol oxidase (*cydAB*) and nitrate/nitrite reductase (*nar/nas*).

The acid stress response of *B. cereus* could be divided into four groups (i) general stress response (ii) pH homeostasis, (iii) metabolic rearrangements and (iv) secondary oxidative stress response (**Figure 6**). The current knowledge, presented in this review, may then lead to the identification of indicators or biomarkers of bacterial behavior. Indeed the finding of molecular biomarkers to characterize the bacterial physiological state under specific conditions remains a key issue for food industry (Kort et al., 2008; den Besten et al., 2010, den Besten et al., 2013; Desriac et al., 2012, 2013). Actually, the combination of molecular tools and predictive microbiology concepts appears as an interesting challenge for food formulation and preservation optimization (McMeekin et al., 2008; Havelaar et al., 2010; Rantsiou et al., 2011; Brul et al., 2012; Desriac et al., 2012).

## Conflict of Interest Statement

The authors declare that the research was conducted in the absence of any commercial or financial relationships that could be construed as a potential conflict of interest.
